# Immunostimulant potential of *Moringa Oleifera* leaves alcoholic extract versus Oregano Essential Oil (OEO) against cyclophosphamide-induced immunosuppression in broilers chicks

**DOI:** 10.1007/s11250-023-03620-5

**Published:** 2023-05-19

**Authors:** Mostafa Abdelgaber Mohamed, Amany Abdelbaky El-Mleeh, Rania Talat Hamad, Ibrahim Said Abu-Alya, Mohamed Hamdy El-Hewaity, Ahmed Ragab Elbestawy, Abdelrahman Mahmoud Elbagory, Ahmed Saber Sayed-Ahmed, Mabrouk Attia Abd Eldaim, Omnia Ibrahim Elshabrawy

**Affiliations:** 1 Department of Pathology, Faculty of Veterinary Medicine, Menoufia University, Shebeen Elkom, 32511 Egypt; 2 Department of Pharmacology, Faculty of Veterinary Medicine, Menoufia University, Shebeen Elkom, 32511 Egypt; 3 Department of Physiology, Faculty of Veterinary Medicine, University of Sadat City, Sadat City, 23897 Egypt; 4 Department of Poultry and Fish Diseases, Faculty of Veterinary Medicine, Damanhour University, El Beheira, 22511 Egypt; 5 Department of Food Hygiene and Control, Veterinary Medicine, Menoufia University, Shebeen Elkom, 32511 Egypt; 6 Department of Anatomy and Embryology Faculty of Veterinary Medicine, Menoufia University, Shebeen Elkom, 32511 Egypt; 7 Department of Biochemistry and Chemistry of Nutrition, Faculty of Veterinary Medicine, Menoufia University, Shebeen Elkom, 32511 Egypt

**Keywords:** *Moringa oleifera*, Oregano Essential Oil, Cyclophosphamide

## Abstract

The current study was conducted to evaluate the immunoenhancement effect of *Moringa oleifera* leaves alcoholic extract (MOLE) versus Oregano essential oil (OEO) against cyclophosphamide induced immunosuppression in broilers chicks. A total of a three hundred one-day-old chicks were assigned randomly into three main dietary groups, control, MOLE, and OEO for 14 days. After 14 days the three main experimental groups were subdivided into six groups, control, cyclophosphamide, MOLE, MOLE and Cyclophosphamide, OEO, and OEO and cyclophosphamide. Each group of these six groups was subdivided into three subgroups. Supplementation of broiler chicks with MOLE and OEO for 14 days significantly increased body weight compared to the control group. However, injection of broiler chicks with cyclophosphamide significantly induced body weight loss, impaired immunological response represented by decreasing total leukocytic count, differential leukocytic count, phagocytic activity, phagocytic index, and hemagglutinin inhibition titer for New Castle disease virus, lymphoid organs depletion, and increased the mortality rate. In contrast, supplementation of cyclophosphamide treated chicks with MOLE and OEO significantly reduced cyclophosphamide induced body weight loss and impaired immunological responses, as it showed significant increase in body weight, total leukocytic count, differential leukocytic count, phagocytic activity, phagocytic index, and hemagglutinin inhibition titer for New Castle disease virus, lymphoid organs proliferation, and reduced the mortality rate. This study indicated that MOLE and OEO supplementation ameliorated cyclophosphamide induced body weight loss and impaired immunological responses.

## Introduction

Poultry farming is essential for the socioeconomic development of underdeveloped nations because hens are a practical and economical source of animal protein (Olwande et al. 2010; Melesse et al. 2013). When a bird's immune system is weakened, infectious pathogens like bacteria, viruses, parasites, and fungi can readily infect it. This can result in a variety of complicated infections (Paliwal et al. 2011). When the body's immune response is reduced due to pharmacological or environmental factors, this is known as immunosuppression (Choudhary 2015). White blood cell counts are one of the immunosuppression indicators in an animal model because a change in the blood's cellular composition is one of the events that trigger immunosuppression (Al-Fararjeh et al. 2013).

A popular alkylating medication called cyclophosphamide is used to treat many diseases like lymphoma, myeloma, and chronic lymphocytic leukemia (Sultana et al. 2011). It is a powerful immunosuppressive drug that crosslinks the DNA of dividing cells. However, its use was limited by its immunosuppressive effects on both cellular and humoral immune responses (Oger 2007) leading to a variety of illnesses (Kajaria et al. 2013). High dose of cyclophosphamide is used to eliminate malignant hematopoietic cells because of its immunosuppressive effects as it alters the secretion of Th2 cytokines like IL-4 and IL-10 while decreasing interferon-gamma and IL-12 in the cerebrospinal fluid and peripheral circulation (Chatelanat et al. 2018). Cyclophosphamide is considered as immune suppressive compound in chicken as it induces sever atrophy of the main lymphoid organs, Bursa of Fabricius, thymus, and spleen. In addition, it induces severe necrosis and lymphocytic depletion in the Bursa of Fabricius, spleen and thymus, lymphopenia, hemorrhages and ulceration in caecal tonsils (Igwe et al. 2018). Thus, cyclophosphamide is used to investigate the immunomodulatory effects of plant extract as it has some immunosuppressive potential (Attia et al. 2014).

There is a great interest in the development of superior immunostimulant compounds derived from natural sources to promote stronger immune responses (Naved et al. 2005).

*Moringa Oleifera* leaves can be utilized in chicken nutrition. (Melesse et al. 2013; Tesfaye et al. 2013) to increase poultry production effectiveness since it is devoid of heavy metals and rich in vital nutrients such protein, beta-carotene (provitamin A), vitamin K, manganese, vitamin C, and vitamin B complex (Donkor et al. 2013; Leone et al. 2015). Moreover, it also has a high concentration of antioxidants such as carotenoids, tocopherols, and ascorbic acid (Qwele et al. 2013; Saini et al. 2014). To date, many *invitro* studies have demonstrated the stimulatory effect of *Moringa Oleifera* on the immune system (Gupta et al. 2018). Moreover, extracts from *Moringa Oleifera* sections are rich in quercetin and kaempferol glucosides, that have been shown to have anti-inflammatory activities (Maheshwari et al. 2014; Stohs and Hartman 2015). Although the main mechanism of action of *Moringa Oleifera* leaves to stimulate the humoral and cellular immunity still unclear, a mice model showed that chronic administration of *Moringa Oleifera* leaves extract significantly increased white blood cell (WBC) count and percentage of neutrophils (Gupta et al. 2010).

Essential oils can enhance the immune response, gut health, antioxidant activity and blood. Also, they reduce serum cholesterol level in broiler chicks (Chowdhurya et al. 2018). A well-known culinary spice and herbal remedy is oregano. It can also be added to poultry feed as a supplement to boost development and immunity (Alagawany et al. 2018). Moreover, oregano is used to improve the organoleptic properties of meals. The oregano leaves are used to extract oregano essential oil (OEO), which is rich in thymol and carvacrol (Figiel et al. 2010). Oregano essential oil promotes faster growth and reduce the utilization of antibiotics in broiler chicks (Symeon et al. 2010), as it contains high concentrations of phytonutrients such antioxidants carotenoids, tocopherols, and ascorbic acid (Qwele et al. 2013; Saini et al. 2014). Therefore, the present study was conducted to evaluate the immunostimulant effect of *Moringa oleifera* leaves ethanolic extract (MOLE) versus Oregano essential oil against cyclophosphamide induced immunosuppression in broilers chicks.

## Material and methods

### Experimental chicks and feeding

This experiment was approved and carried out in accordance with the ethical guidelines of the Animal Care and Use Committee of Faculty of Veterinary Medicine, University of Menoufia, Egypt.

Three hundred one-day-old, unsexed broiler chick were randomly divided into three main groups,100 chicks each.

#### Control group

Chicks were given drinking water and basal diet without additives.

#### The second group

Chicks were supplemented with MOLE at a dose of 200 mg/L in drinking water (Hamada et al. 2021).

#### The third group

Chicks were given Oregano essential oil (OEO) at a dose of 100 mg/L in drinking water.

Each group was divided into three sub-groups for 14 days. On 14 ^th^ day old the three main group were subdivided into six groups, 50 chicks each as the following:

- **Control group**: Chicks were given drinking water and a standard diet without additives till the end of the experiment (35 day old).
- **Cyclophosphamide group**: Chicks were given a standard diet and water without additives from one day old and were injected with cyclophosphamide at a dose of 75 mg/kg body weight/day in the thigh muscle for three successive days (14^th^ , 15^th^ and 16^th^ day of age) (Igwe et al. 2019) and given drinking water and standard diet without additives till the end of the experiment.
- ***Moringa Oleifera leaves ethanolic extract (MOLE) group***: Chicks were supplied with a standard diet and MOLE at a dose of 200 mg/L in drinking water from till the end of the experiment (Hamada et al. 2021).
- ***Moringa oleifera leaves ethanolic extract and Cyclophosphamide group***: Chicks were supplemented with a standard diet and MOLE at a dose of 200 mg/L of drinking water till the end of the experiment and were injected with cyclophosphamide at a dose of 75 mg/kg body weight in the thigh muscle for three successive days (14^th^ , 15^th^ and 16^th^ day of age).
- **Oregano essential oil group**: Chicks were supplied with a standard diet and OEO at a dose of 100 mg/L in drinking water till the end of the experiment.
- **Oregano essential oil and Cyclophosphamide group**: Chicks were supplemented with a standard diet and OEO at a dose of 100 mg/L in drinking water from till the end of the experiment and were injected with cyclophosphamide at a dose of 75 mg//kg body weight in the thigh muscle for three successive days (14^th^ , 15^th^ , and 16^th^ day of age).

Each group of these six groups was divided into three subgroups.

### Ration and additives

The chicks were fed a starter ration for 12 days of age, while they were given a grower ration from 12^th^ to 22^nd^ day of age then, they were fed on a finisher ration from 23^rd^ day of age till the end of the experiment (35^th^ day of age) (Table 1). Chicks were allowed for food and water *add libtuim*.

**Table 1:** the composition of broiler diets

Ingredient and composition g/100 g of ration	Starter	Grower	Finisher
Corn	57.89	61.26	62.4
Soybean meal 48%	35.00	31.00	29.00
Soybean oil	2.40	3.20	4.50
Monocalcium phosphate	1.30	1.15	1.00
Limestone	1.50	1.50	1.45
DL-Methionine	0.35	0.33	0.30
L-Lysine (%)	0.44	0.37	0.28
Threonine	0.18	0.23	0.23
Tryptophan	0.00	0.01	0.17
L-Valine	0.10	0.11	0.08
NaCl	0.23	0.24	0.23
Choline Chloride (%)	0.10	0.10	0.10
Trace mineral permix^1^	0.10	0.10	0.10
Vitamin permix^2^	0.10	0.10	0.10
Sodium sulphate	0.26	0.25	0.23
Ronozyme NP^3^	0.02	0.02	0.02
Rovabio Excel AP^4^	0.01	0.01	0.01
Ronozyme ProAct^5^	0.02	0.02	0.02
Antioxidant	0.01	0.01	0.01

The experiment was conducted with a 14-hour light/ten-hour dark cycle at ambient temperature. The room was maintained at a constant temperature of 23–25 °C and a relative humidity of 50–70 % during the experiment. Throughout the trial, the birds were maintained in constant climatic and nutritional conditions. Infectious Bronchitis Disease (IBD) and New castle Disease (ND) vaccinations were administered to chicks according to the recommended timetable.

### Body weight gain (g)

By subtracting the initial body weight at the start of the experiment from the final body weight on day 35.

### Collection of blood samples

On days 17^th,^ 21^st^, 28^th^ and 35^th^ of age, blood samples were collected from the wing vein of all groups. Whole blood samples were collected in tubes containing anticoagulant for determination of total leukocytic count, differential leukocytic count, phagocytic activity, and phagocytic index. While other blood samples were collected without anticoagulant and incubated for 1h at room temperature for coagulation, centrifugated for 15 mins. at 3500 rpm then clear sera were separated and used for determination of Hemagglutinin inhibition (HI) testing for New Castle Disease Virus (NDV).

### Collection of tissue samples

Spleen and bursa samples were collected, preserved in 10% buffered neutral formalin and processed according to Bancroft and Stevens (1996).

### Determination of phagocytic activity % and phagocytic index of heterophils

Phagocytic activity was performed using Candida albicans according to the method described by Sornplang et al. (2015). Briefly, 100 μl of broiler chick serum, 100 μl of gently collected blood sample, and 100 μl of heat-killed Candida albicans (5 X 106/ml) were mixed, incubated at 37 ºC for 30 mins and centrifuged at 1000 rpm for 5 mins. Blood smears were done, air dried, fixed with pure methyl alcohol, and stained with Giemsa. One hundred heterophils were examined and the percentage of heterophils ingesting Candida was calculated to determine the phagocytic activity. The phagocytic index is computed by dividing the amount of engulfed Candida by the number of the active heterophils.

$$\mathrm{Phagocytic \ Activity \%}=\frac{\mathrm{No \ of \ heterophils \ ingesting \ }candida}{\mathrm{Total \ number \ of \ heterophils}}\times 100$$

$$\mathrm{Phagocytic \ index}=\frac{\mathrm{Total \ Number \ of \ ingested \ }candida}{\mathrm{Number \ of \ active \ heterophils}}$$

### Determination of humoral immune response for the Newcastle disease vaccines using hemagglutination Inhibition (HI) test

Blood samples from each group (*n* = 5) were collected from the wing vein on days 17th, 21st, 28th and 35^th^ of age without anticoagulant for serum separation. Clear serum samples were separated and used for Hemagglutination inhibition (HI) test against NDV. The hemagglutination (HA) units for standard viral antigens [NDV (LaSota strain) were 8 log2 (Office International des Epizooties (OIE) 2021).

### Statistical analysis

Data were presented as means ± SE. Statistical analysis was performed using one-way ANOVA followed by Duncan’s multiple range tests to detect the significance of differences. The level of statistical significance was set at P≤0.05.

## Results

### Body weight responses of broiler chicks

Supplementation of broiler chicks with MOLE and OEO in drinking water from the beginning of the experiment till 14 days of age significantly increased final chicks body weight (19.4% and 9.94% respectively) compared with those of the control chicks (p ≤ 0.01) (Table 2). In addition, supplementation of broiler chicks with MOLE and OEO till the end of the experiment increased final body weights by 9.1% and 6.4% respectively, compared with the control group (Table 3).

**Table 2:** Effects of MOLE and Oregano on body weight after 14 days from beginning of experiment

Parameter	Control	MOLE	Oregano
14^th^ day body weight (g)	313.82 ± 4.38^b^	374.67 ± 6.43^a^	345 ± 4.12^a^

Values are expressed as means ± standard error of the mean

Values carrying different superscript letters within each row are significantly different

**Table 3:** Effect of MOLE and Oregano against Cyclophosphamide on the final body weight of broiler chicks

Parameter	Control	Cyclo	MOLE	MOLE & cyclo	Oregano	Oregano & cyclo
Final body weight (g)	1849 ± 53.4^a^	1555 ± 42.37^c^	2019 ± 38.18^a^	1650 ± 35.62^b^	1970 ± 50.26^a^	1662 ± 39.48^b^

Values are expressed as means ± standard error of the mean

Values carrying different superscript letters within each row are significantly different

Injection of chicks with cyclophosphamide on 14^th^ day of age significantly decreased final body weight compared with those of the control chicks (Table 2). However, supplementation of cyclophosphamide administrated chicks with MOLE or OEO significantly increased final body weights (6.1% and 6.88% respectively) compared with those of cyclophosphamide group (p ≤ 0.05) (Table 3).

### Cell mediated immunity of the broiler chick

Administration of broiler chicks with Cyclophosphamide at 14^th^ day of age significantly decreased total leukocytic count, differential leukocytic count, phagocytic activity, and phagocytic index from 17^th^ day of the age till the end of the experiment compared to the control group. However, administration of chicks with MOLE increased above mentioned parameters from 21^1st^ till the end of the experimental period compared to the control group. In addition, administration of broiler chicks with OEO significantly increased the total leucocytic count on day 21^1st^ day of age, heterophils count, phagocytic activity, and phagocytic index all over the experimental period compared to the control group. Moreover, supplementation of broiler chicks administrated cyclophosphamide with MOLE and OEO significantly increased total leukocytic count, differential leukocytic count, phagocytic activity, and phagocytic index from 17^th^ day of the age till the end of the experiment compared to the Cyclophosphamide group (Table 4).

**Table 4:** Effects of MOLE, Oregano and/or Cyclophosphamide on total leukocytic count, differential leukocytic count, phagocytic activity, and phagocytic index

Parameter	Control	Cyclopho	MOLE	MOLE & Cyclo	Oregano	Oregano & cyclo
Total leukocytic count 10^3^/mm3) 17^th^ day old	20.58 ± 0.92^ab^	14.62 ± 0.89^c^	22.17 ± 1.25^a^	19.39 ± 1.07^ab^	22.08 ± 1.14^a^	18.62 ± 0.57^b^
21^1st^ day	20.74 ± 0.81^b^	14.13 ± 0.99^d^	23.47 ± 1.02^a^	20.74 ± 0.58^b^	23.76 ± 0.82^a^	18.00 ± 0.71^c^
28^th^ day	22.46 ± 0.96^b^	17.02 ± 0.80^c^	28.25 ± 0.67^a^	23.23 ± 1.08^b^	24.11 ± 1.02^b^	22.00 ± 0.46^b^
35^th^ day	22.67 ± 0.64^b^	19.13 ± 1.04^c^	28.82 ± 1.08^a^	24.98 ± 1.14^b^	25.00 ± 0.97^b^	22.88 ± 0.41^b^
Heterophils count (10^3^/mm^3^) 17^th^ day	4.96 ± 0.22^b^	3.13 ± 0.19^c^	6.58 ± 0.37^a^	4.89 ± 0.27^b^	5.83 ± 0.30^a^	4.45 ± 0.14^b^
21^1st^ day	4.94 ± 0.19^ cd^	3.09 ± 0.22^e^	7.09 ± 0.31^a^	5.41 ± 0.15^c^	6.23 ± 0.22^b^	4.32 ± 0.17^d^
28^th^ day	5.53 ± 0.24^c^	3.780 ± 0.18^d^	8.62 ± 0.21^a^	6.34 ± 0.29^b^	6.53 ± 0.28^b^	5.33 ± 0.11^c^
35^th^ day	5.49 ± 0.16^c^	4.28 ± 0.23^d^	9.02 ± 0.34^a^	7.04 ± 0.32^b^	7.08 ± 0.28^b^	5.72 ± 0.10^c^
Lymphocytes count (10^3^/mm^3^) 17^th^ day	13.85 ± 0.62^a^	10.26 ± 0.63^b^	14.21 ± 0.80^a^	12.87 ± 0.71^a^	14.60 ± 0.75^a^	12.57 ± 0.39^a^
21^1st^ day	14.12 ± 0.55^ab^	9.85 ± 0.69^d^	14.88 ± 0.65^ab^	13.52 ± 0.38^bc^	15.45 ± 0.53^a^	12.24 ± 0.48^c^
28^th^ day	15.03 ± 0.64^b^	11.89 ± 0.56^c^	17.86 ± 0.43^a^	15.05 ± 0.70^b^	15.58 ± 0.66^b^	14.74 ± 0.31^b^
35^th^ day	15.21 ± 0.43^b^	13.41 ± 0.73^c^	18.16 ± 0.68^a^	16.11 ± 0.73^b^	16.05 ± 0.62^b^	15.26 ± 0.27^b^
Phagocytic activity (%) 17^th^ day	46.08 ± 1.64^c^	37.89 ± 0.79^d^	64.26 ± 0.96^a^	54.95 ± 0.64^b^	54.47 ± 0.79^b^	46.47 ± 0.87^c^
21^1st^ day	49.73 ± 0.41^d^	34.22 ± 0.63^e^	66.24 ± 0.91^a^	61.25 ± 0.66^b^	61.66 ± 0.57^b^	52.28 ± 0.63^c^
28^th^ day	51.04 ± 0.68^c^	34.97 ± 1.06^d^	70.24 ± 0.64^a^	64.03 ± 0.85^b^	64.76 ± 0.91^b^	52.67 ± 0.82^c^
35^th^ day	51.94 ± 0.61^d^	39.13 ± 0.98^e^	70.82 ± 0.91^a^	67.34 ± 0.82^b^	67.01 ± 0.89^b^	56.38 ± 0.65^c^
Phagocytic index 17^th^ day	1.82 ± 0.064^c^	1.52 ± 0.044^d^	2.38 ± 0.074^a^	2.15 ± 0.50^b^	2.11 ± 0.045^b^	1.62 ± 0.057^d^
21^1st^ day	1.79 ± 0.041^d^	1.51 ± 0.041^e^	2.52 ± 0.066^a^	2.15 ± 0.049^b^	2.23 ± 0.065^b^	1.94 ± 0.037^c^
28^th^ day	1.81 ± 0.057^c^	1.37 ± 0.049^d^	2.65 ± 0.066^a^	2.26 ± 0.049^b^	2.35 ± 0.065^b^	1.96 ± 0.081^c^
35^th^ day	1.88 ± 0.036^d^	1.57 ± 0.065^e^	2.74 ± 0.089^a^	2.54 ± 0.095^ab^	2.52 ± 0.059^b^	2.19 ± 0.060^c^

Values are expressed as means ± standard error of the mean

Values carrying different superscript letters within each row are significantly different (*P* ≤ 0.05)

### Immune response of the broiler chick to New Castle disease vaccine

Administration of broiler chicks with cyclophosphamide numerically decreased the HI titer of chicks from 17^th^ till 28^th^ days of age and significantly decreased HI titer on 35^th^ day of the age compared to the control group. However, supplementation of cyclophosphamide administrated chicks with MOLE and OEO normalized the HI titer all over the experimental period compared cyclophosphamide treated group. The cyclophosphamide group had the lowest significant HI titer for ND compared to all other groups while chicks administrated with cyclophosphamide and supplemented with MOLE had the highest (Table 5).

**Table 5:** Effects of MOLE, Oregano and/or Cyclophosphamide on hemagglutinin inhibition (HI) testing for New Castle disease virus (NDV)

Sampling	Control	Cyclophosphamide	MOLE	MOLE & cyclo	Oregano	Oregano & cyclo
17^th^ day old	0.67 ± 0.67	0.33 ± 0.33	1.17 ± 0.83	1.33 ± 0.99	0.67 ± 0.49	1.50 ± 0.56
21^st^ day old	4.83 ± 1.08	2.83 ± 0.31	4.33 ± 0.49	4.50 ± 0.76	3.67 ± 0.61	3.50 ± 0.34
28^th^ day old	9.33 ± 0.76	8.67 ± 0.21	8.67 ± 0.21	8.83 ± 0.31	8.17 ± 0.40	7.83 ± 0.79
35^th^ day old	8.33 ± 0.56^ab^	7.67 ± 0.33^b^	8.33 ± 0.33^ab^	9.17 ± 0.40^a^	8.67 ± 0.42^ab^	8.67 ± 0.49^ab^

Values are expressed as means ± standard error of the mean

Values carrying different superscript letters within each row are significantly different (*P* ≤ 0.05)

### Lymphoid organ of broiler chicks

**Fig. 1:**
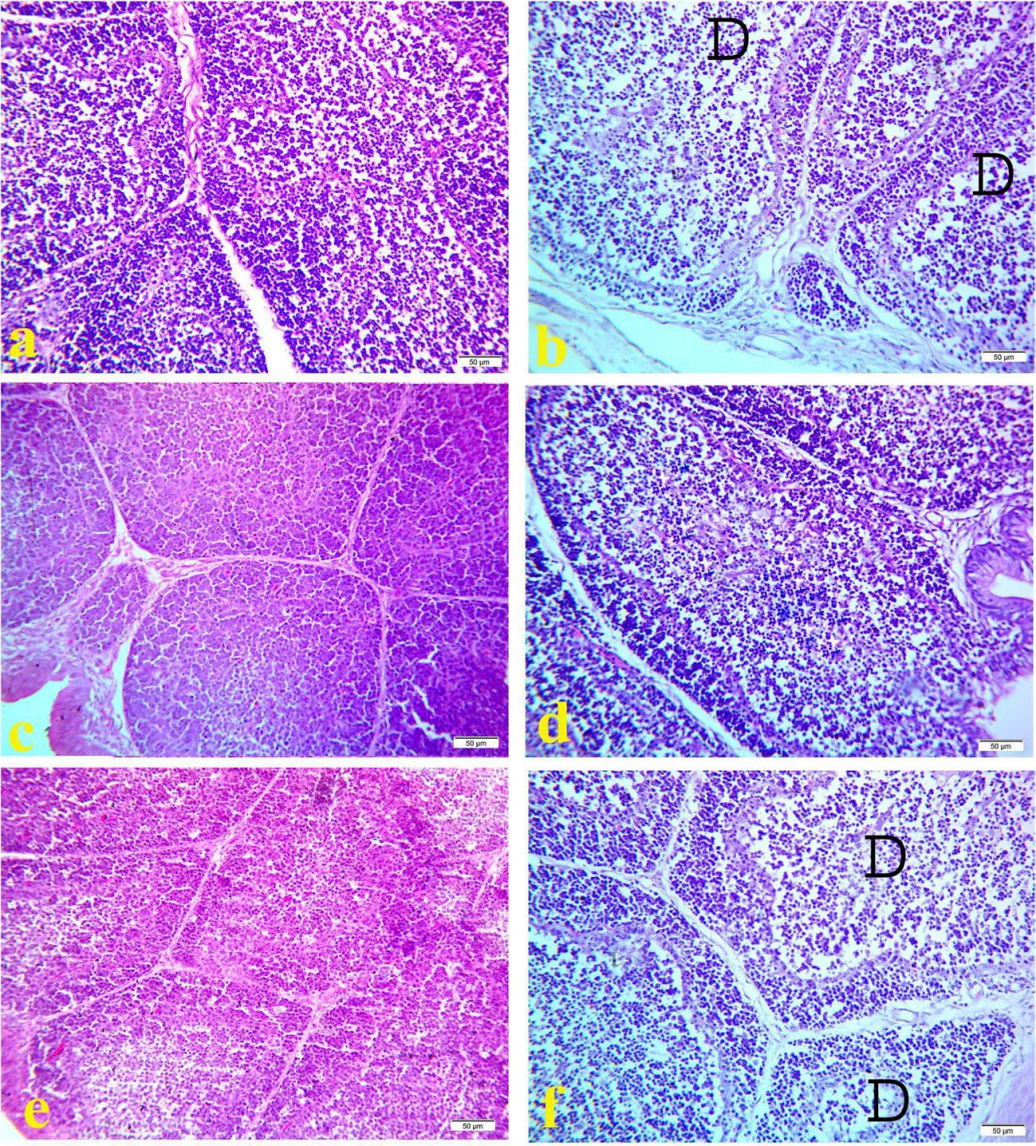
Histopathology of bursa of Fabricious at 17 days old chicks: **a** control group; **b** cyclophosphamide treated group; **c** MOLE treated group; **d** MOLE and cyclophosphamide treated group; **e** OEO treated group; **f** OEO and cyclophosphamide treated group. Where D indicates lymphoid depletion. H&E. 40X. Bar is 50 μm

**Fig. 2:**
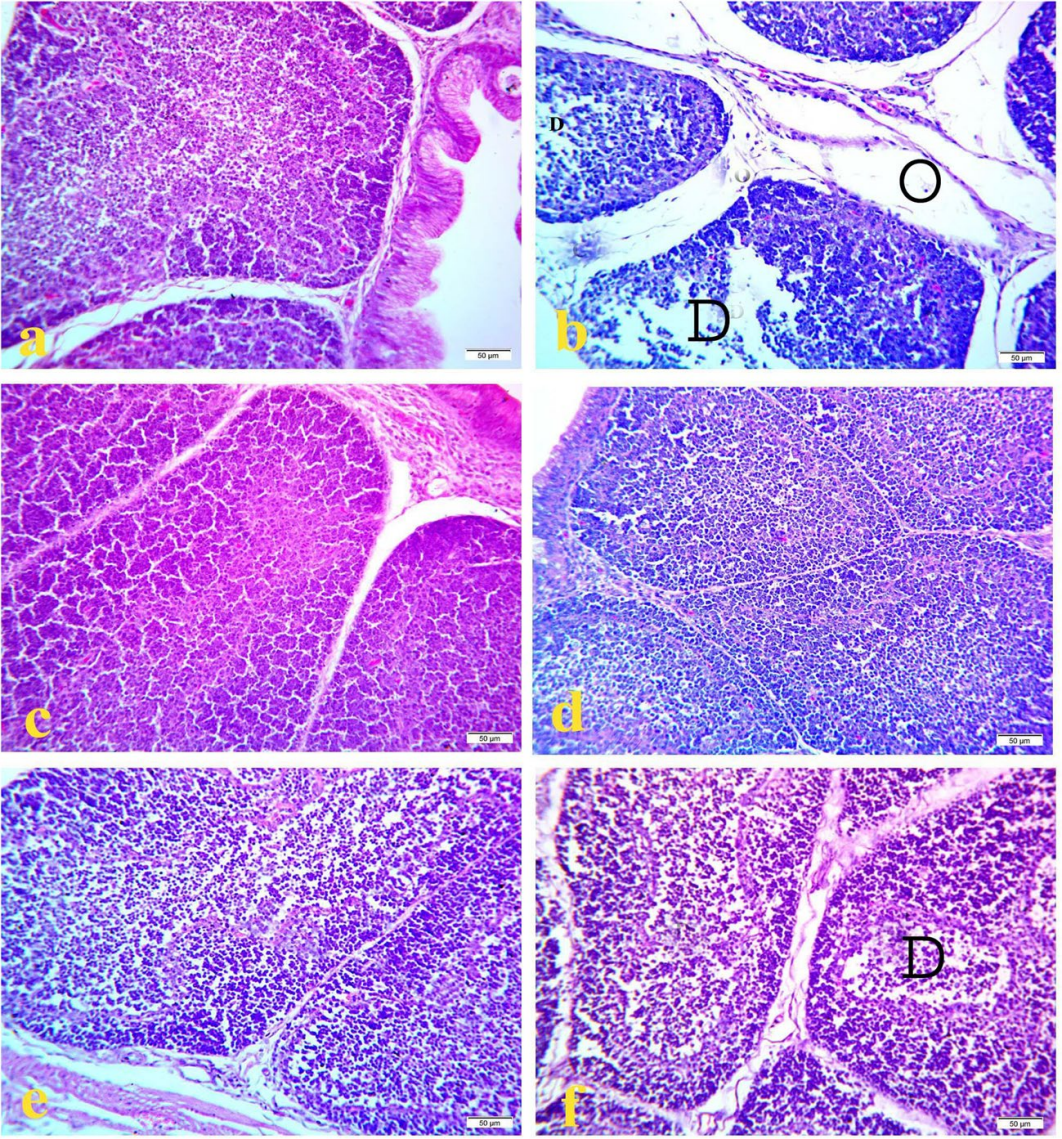
Histopathology of bursa of Fabricious at 21 days old chicks: **a** control group; **b** cyclophosphamide treated group; **c** MOLE treated group; **d** MOLE and cyclophosphamide treated group; **e** OEO treated group; **f** OEO and cyclophosphamide treated group. Where D indicates lymphoid depletion, O indicate edema, inflammatory cell (arrow). H&E. 40X. Bar is 50 μm

**Fig. 3:**
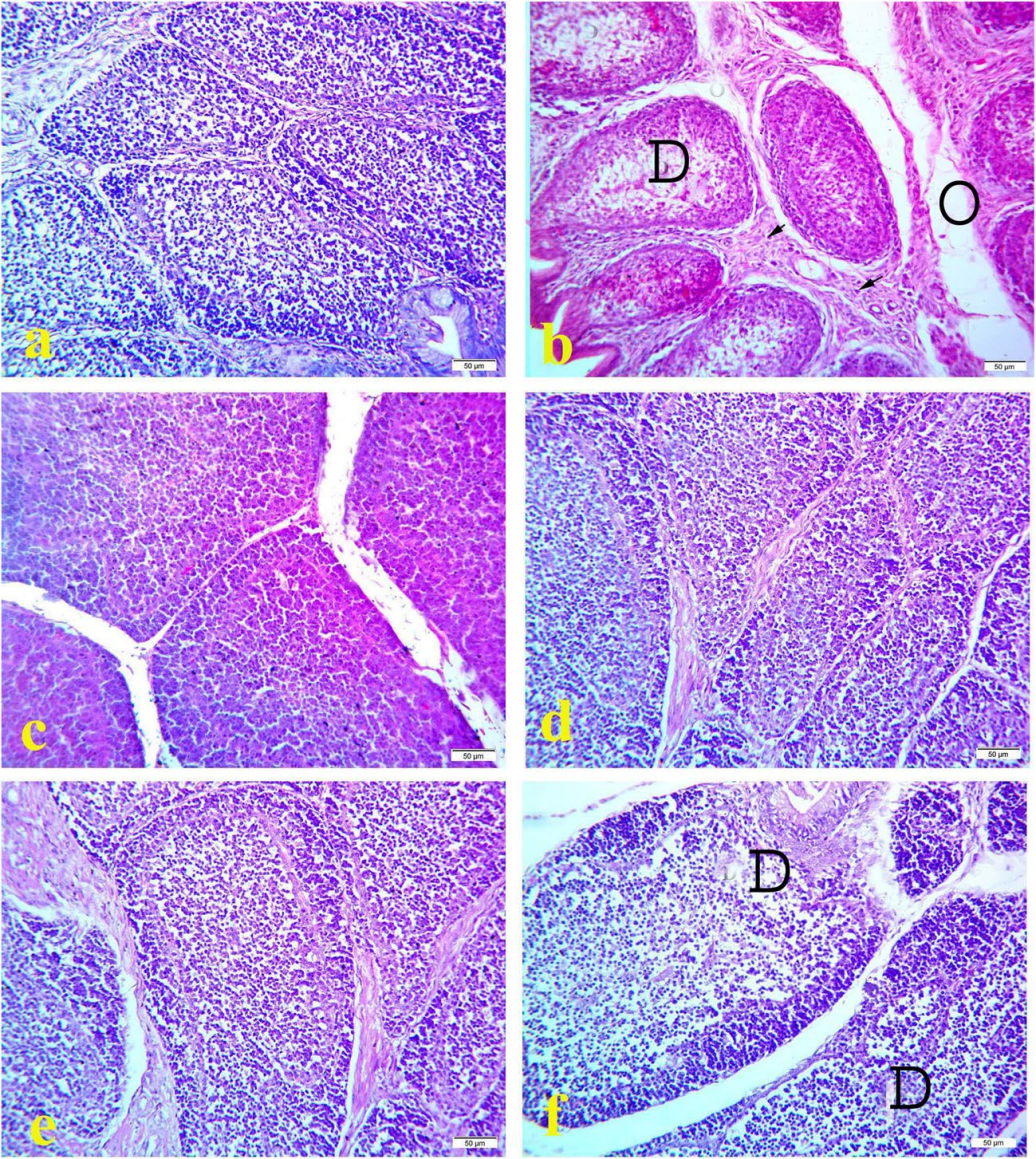
Histopathology of bursa of Fabricious at 28 days old chicks: **a** control group; **b** cyclophosphamide treated group; **c** MOLE treated group; **d** MOLE and cyclophosphamide treated group; **e** OEO treated group; **f** OEO and cyclophosphamide treated group. Where D indicates lymphoid depletion. H&E. 40X. Bar is 50 μm

Histopathological examination of bursa of fabricious of the control (Figs. 1a, 2a, and 3a) and OEO treated groups (Figs. 1e, 2e, and 3e) showed normal lymphoid follicles structure during the experimental period. However, the bursa of `fabricious of cyclophosphamide treated group showed mild to moderate depletion of lymphoid cells from center of the bursal follicles at 17^th^ day of age (3 days after the first injection of cyclophosphamide) **(**Fig. 1b). On 21^1st^ day old (7 days after the first injection of cyclophosphamide) bursa showed distended interfollicular spaces with edematous fluid and reactive cells infiltration, mild interfollicular fibrosis, moderate follicular atrophy, and moderate depletion of lymphoid cells from center of the bursal follicles (Fig. 2b). On the 28^th^ day old (2 weeks days after the first injection of cyclophosphamide) bursa showed marked interfollicular fibrosis with reactive cells infiltration, mild interfollicular edema, severe follicular atrophy, and severe depletion of lymphoid cells from center of the bursal follicles (Fig. 3b). On the contrast bursa of MOLE treated group showed hyperplasia of lymphoid cells of the bursal follicles during the experimental period (Figs. 1c, 2c, and 3c). While the bursa of MOLE and cyclophosphamide treated group showed protection against the depletion of lymphoid cells of the bursal follicles caused by cyclophosphamide and the lymphoid follicles appear nearly normal along the experimental duration (Figs. 1d, 2d, and 3d). The bursa of OEO and cyclophosphamide treated group showed mild to moderate depletion of lymphoid cells from the center of the bursal follicles along the experimental duration (Figs. 1f, 2f, and 3f)

**Fig. 4:**
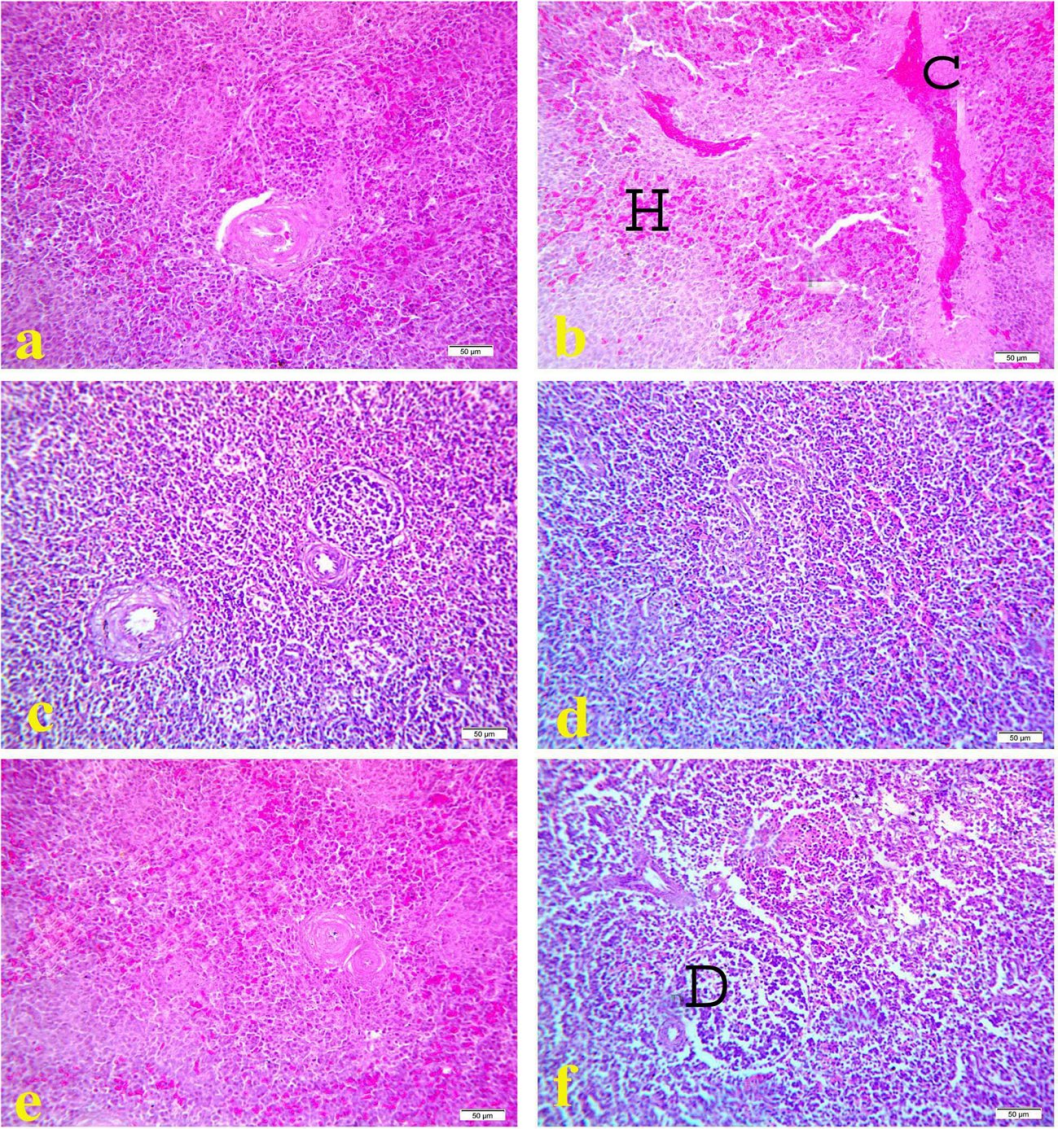
Histopathology of spleen at 17 days old chicks: **a** control group; **b** cyclophosphamide treated group; **c** MOLE treated group; **d** MOLE and cyclophosphamide treated group; **e** OEO treated group; **f** OEO and cyclophosphamide treated group. Where D indicates lymphoid depletion, C indicates congestion of splenic sinus, H indicates area of hemorrhage. H&E. 40X. Bar is 50 μm

**Fig. 5:**
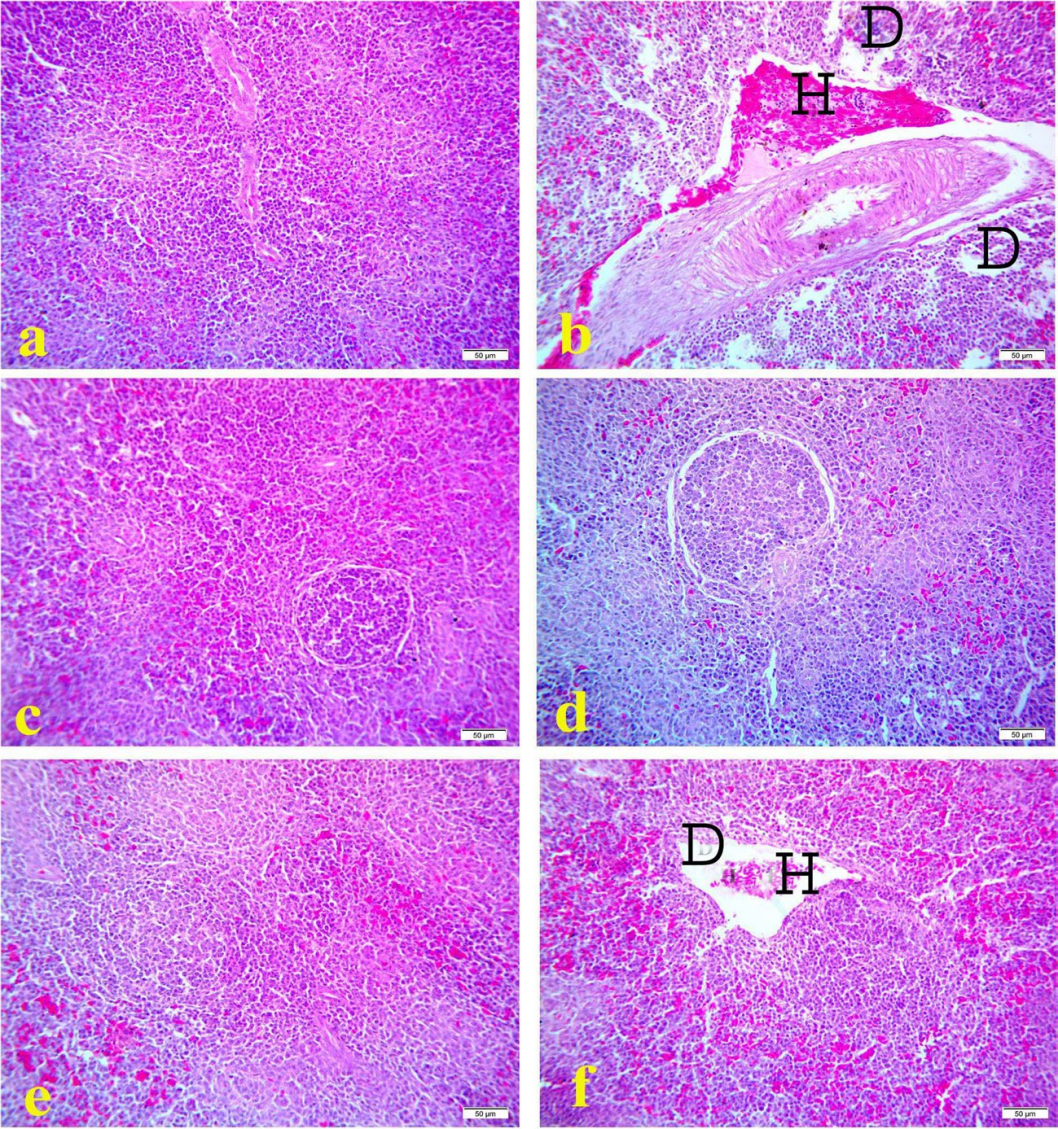
Histopathology of spleen at 21 days old chicks: **a** control group; **b** cyclophosphamide treated group; **c** MOLE treated group; **d** MOLE and cyclophosphamide treated group; **e** OEO treated group; **f** OEO and cyclophosphamide treated group. Where D indicates lymphoid depletion, C indicates congestion of splenic sinus, H indicates hemorrhage. H&E. 40X. Bar is 50 μm

**Fig. 6:**
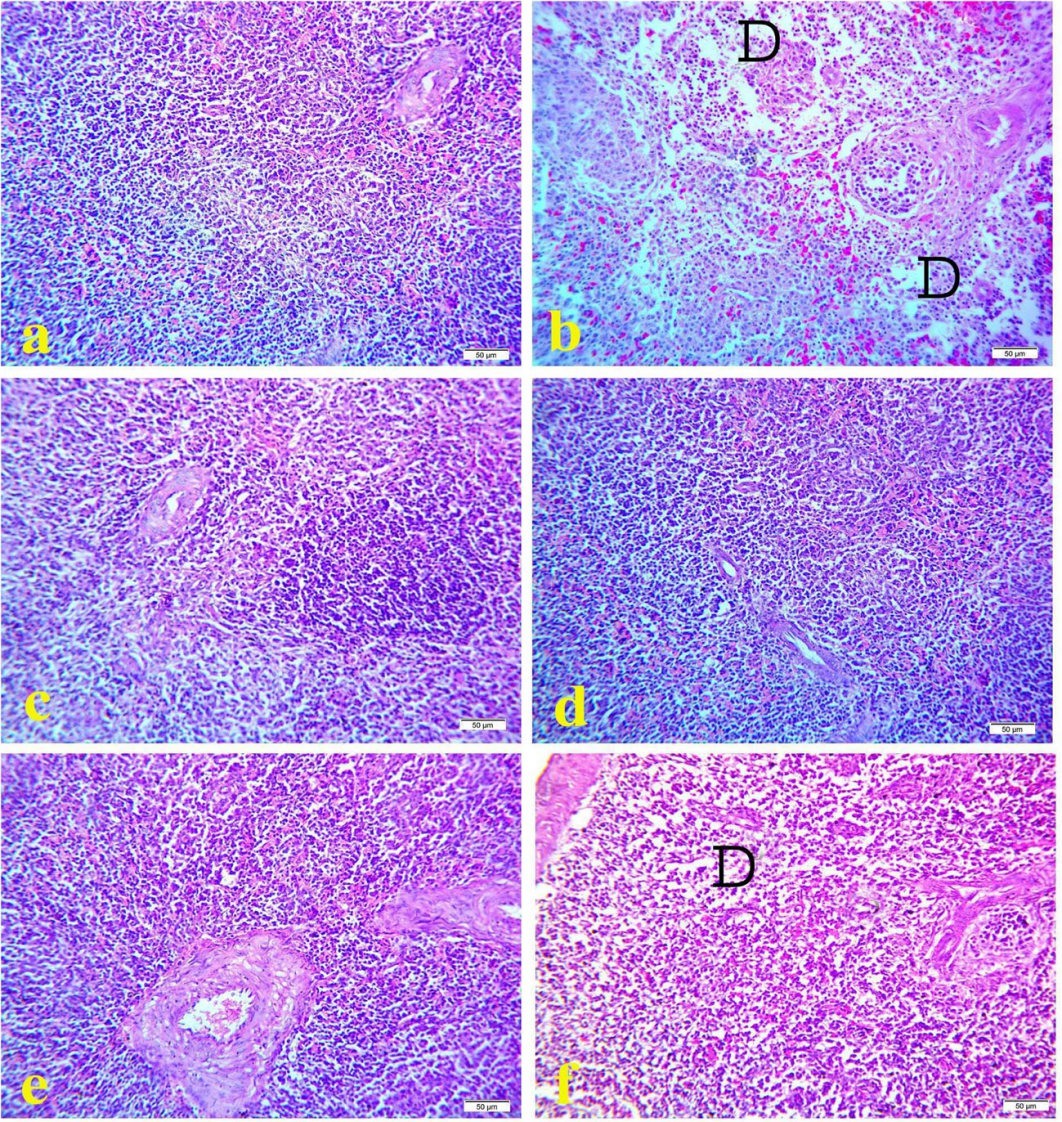
Histopathology of spleen at 28 days old chicks: **a** control group; **b** cyclophosphamide treated group; **c** MOLE treated group; **d** MOLE and cyclophosphamide treated group; **e** OEO treated group; **f** OEO and cyclophosphamide treated group. Where D indicates lymphoid depletion, C indicates congestion of splenic sinus, H indicates hemorrhage. H&E. 40X. Bar is 50 μm

Histopathological examination of spleen of the control group (Figs. 4a, 5a, and 6a) and OEO treated group (Figs. 4e, 5e, and 6e) showed normal histological pictures during the experimental period. However, spleen of cyclophosphamide treated group showed congestion of splenic sinus with area of hemorrhage on17^th^ day old (Fig. 4b) while on 21^1st^ day-old spleen showed severe lymphoid depletion with severe area of hemorrhage (Fig. 5b). On 28^th^ day old the spleen samples showed severe lymphoid depletion (Fig. 6b). On the contrast, along the experimental duration spleen samples of MOLE treated group showed hyperplasia of lymphoid follicles (Figs. 4c, 5c, and 6c). While spleen of MOLE and cyclophosphamide treated group appeared nearly normal structure and protected against lymphoid depletion caused by cyclophosphamide (Figs. 4d, 5d, and 6d). Spleen samples from OEO and cyclophosphamide treated group showed mild to moderate lymphoid depletion with area of hemorrhage during the experiment duration (Figs. 4f, 5f, and 6f).

### Mortality rate

Supplementation of broiler chicks with MOLE and OEO reduced the mortality rate by 50%. Administration of chicks with cyclophosphamide led to death of 40% of chicks however, supplementation of cyclophosphamide with MOLE or OEO reduced the mortality rates to 4 % and 10% respectively (Table 6).

**Table 6:** Effects of MOLE, Oregano and/or Cyclophosphamide on mortality rate in broiler chicks:

	Control	Cyclo	MOLE	MOLE and Cyclo	Organo	Organo and cyclo
Total number of chicks	50	50	50	50	50	50
Number of live chicks	48	30	49	48	49	45
Number of dead chicks	2	20	1	2	1	5
Mortality Rate (%)	4	40	2	4	2	10

## Discussion

Humans can get high-quality animal protein from poultry meats and eggs as poultry meat represents about 20% of each person's daily intake of fish and animal proteins (Alders et al. 2018). Long-term exposure to harmful and infectious elements can worsen health, reduce body weight gains, reduce the effectiveness of preventive vaccinations, and raise the risk of developing cancer, parasite infections, and latent infections (Sundar and Sires 2013). Supporting defense system through food and/or herbal treatments can also benefit the gut microbiota, inflammation, viral infections, and nutritional imbalance (Dong et al. 2021). Hence, it is crucial to create immune boosters that are both secure and efficient to battle chicken immunosuppression. Numerous studies have shown that natural products that derived from plants can boost the immune system (Akram et al. 2018). In addition, Essential oils have gained special focus as natural antioxidant and immunostimulant agents. They possess distinctive antimicrobial, antioxidant, anti-inflammatory, anti-stress, appetite stimulators, analgesic, and aphrodisiac activities (Peterfalvi et al. 2019).

The current study revealed that supplementation of broiler chicks with MOLE or OEO for 14 days enhanced chicks body weight and ameliorated cyclophosphamide induced final body weight loss. These results were consistence with those of (Abdulsalam et al. 2015; Alabi et al. 2017; Khan et al. 2017) who mentioned that inclusion of *Moringa Oleifera* and its extract in broiler chicks’ diets improves their final body weights and growth performance. Another study claimed that alkaloid that present in *Moringa* may stimulate the feed consumption in broiler chicks due to their impact on the homeostasis of glucose (Mbikay 2012). The presence of growth-promoting substances and nutritional elements in MOLE, such as carbohydrates, saponins, cardiac glycosides, terpenes, steroids, flavonoids, and alkaloids may be responsible for the enhancement of hens' live body weight (Ambali and Furo 2012). Moreover, MOLE has the ability to increase broiler chick body weight through digestion-enhancing properties that promote the growth of beneficial bacteria and, inhibit the growth of potentially harmful microorganisms (Hernandez et al. 2004).

It has been indicated that supplementation of broiler chicks with OEO improves their growth performance, carcass traits and the health of the intestine (Peng et al. 2016; Zhang et al. 2014). Mathlouthi et al. (2012) suggested that OEO enhances chicken feed because it is rich in thymol and 69.55% carvacol, cymol and monoterpene. These ingredients enable OEO to accelerate growth rate (Nieto et al. 2018), enhance body wight, food conversion ratio, and decrease the mortality rate of broiler chicks (Dafade et al. 2019). Moreover (Zhang et al. 2021) reported that supplementation of broiler chicks with OEO improves their health performance through enhancement of antioxidant statues and intestinal health.

Furthermore, the present study indicated that administration of broiler chicks cyclophosphamide reduced body weight and induced immunosuppression through decreasing total leukocytic count, differential leukocytic count, phagocytic activity, and phagocytic index, and induced lymphoid depletion in bursa of Fabricious and spleen, and consequently increased the mortality rate in chicks. These results were parallel with those of (Yu et al. 2019) who indicated that, cyclophosphamide decreases body weights and induces immunosuppression via reduction of the lymphoid organs weights, atrophic alterations in the lymphoid organs, and changes in NK cell activities. Furthermore, it has been demonstrated that cyclophosphamide enhances the reduction in body weight, immune organ indices, hematological parameters, serum and splenic cytokine levels, and NK cell activities (Kanno et al. 2009). Cyclophosphamide's immunosuppressive properties can be related to its inhibition of hematopoiesis, which lowers white blood cell numbers (Basu et al. 2017). So, it inhibits humoral and cellular immunity (Xie et al. 2016) leading to compromised gut health, immunosuppression, and myelosuppression. All of them have significant negative impact on morbidity and mortality (Shirani et al. 2015).

On the other hand, addition of MOLE to the drinking water of cyclophosphamide treated chicks enhanced their body weight, immune indices, and the proliferation of lymphocytes in their lymphoid organs, which significantly decreased the chick mortality rate. These findings agreed with those of (Gupta et al. 2010) who reported that pretreatment of mice with *Moringa Oleifera* leaf extract prevented the bone marrow suppressive impact of cyclophosphamide on phagocytic activity, which improves both cellular and humoral immunity. In addition, (Ferreira et al. 2009) indicated that *Moringa Oleifera* extract contains numerous minerals, such as calcium ions, so it provides nutritional significance. Moreover, (Madubuike and Ekenyen 2006; Olugbemi et al. 2010 and Oyewo et al. 2012) reported that supplementation of broiler chicks with *Moringa Oleifera* aqueous extract enhances the health of the chicks through enhancing their immune system due to its high content of flavonoids and phenolic substances including quercetin, kaempferol, and rutin. Furthermore, MOLE may have an immunostimulatory effect (Mousa et al. 2019). Additionally, isothiocyanates, glycoside cyanides, and minerals including selenium, manganese, iron, zinc, and magnesium are thought to boost organisms' resistance to disease (Salem 2016). Interestingly, it is demonstrated that Ginseng saponins which have anti-inflammatory, antioxidant, anti-apoptotic, and immune-stimulant properties are also abundant in *Moringa* (Rausch et al. 2006). Furthermore, broiler chickens' total white blood cell, lymphocyte, and immunoglobulin counts are increased by *Moringa Oleifera* extracts (Adedapo et al. 2005). White blood cells have immunomodulatory capabilities as they are involved in battling infection and removing wounded or dead cells and tissues from the body (Oyewo et al. 2012). In the same context, the present study demonstrated that MOLE enhanced phagocytic activity, which may be related to its high mineral and vitamin content, which is crucial to produce numerous cytokines necessary for phagocytic activities (Fakurazi et al. 2008).

The bursa of Fabricious and spleen are crucial immunological organs in chickens. The strength of the immune system is shown by the lymphocyte proliferation, which is determined by the organ index (Wang et al. 2022). The bursa of Fabricious 's T lymphocytes is the main contribution to cellular immunity. The spleen is a peripheral immune organ rich in lymphocytes and macrophages. The level of the immunological organ index can be thought of as the extent of lymphocyte proliferation (Shirani et al. 2015). It has been shown that *Moringa Oleifera* extract has strong antiviral activity against NDV in ovo (Chollom et al. 2012).

Additionally, our findings showed that OEO ameliorated cyclophosphamide induced body weight loss, immunosuppression, depletion of lymphoid organs, and death rate of chicks. These results could be explained by the high concentrations of two monoterpene hydrocarbons; cymol and terpinene, and two phenolic chemicals; carvacrol and thymol, which are found in origanum (Mathlouthi et al. 2012). These phytochemical gradients make it capable of having growth-promoting benefits, antioxidant activity, powerful antibacterial actions against cecal *E. coli* in broilers, and improving broiler immunological response (Roofchaee et al. 2011). Additionally, the presence of thymol and carvacrol in OEO boost the immune responses of the chicks because of its strong antioxidant qualities (Gabor et al. 2010; Feizi and Nazeri 2011). Also, (Hashemipour et al. 2013) reported that thymol and carvacrol can potentially have humoral immunostimulant effects in broilers by raising their total and IgG titers. Furthermore, (Vázquez et al. 2015) demonstrated that levamisole, which is another potent immunostimulant, is not effective in increasing NDV-HI specific Ab titers as Zataria multiflora essential oil (thymol and carvarcol combo).

Finally, the results of the current study indicated that supplementation of broiler chicks administrated cyclophosphamide with MOLE or OEO significantly increased total leukocytic count, differential leukocytic count, phagocytic activity, and phagocytic index from 17^th^ day of the age till the end of the experiment as compared to Cyclophosphamide group and control groups. Also, the histopathological examination of bursa of Fabricious in MOLE and cyclophosphamide treated group showed protective effect against the depletion of lymphoid cells of the bursal follicles caused by cyclophosphamide and the lymphoid follicles appear nearly normal along the experimental duration (Figs. 1d, 2d, and 3d). Furthermore, regarding the spleen of MOLE and cyclophosphamide, the treated group appeared nearly normal structure and protected against lymphoid depletion caused by cyclophosphamide (Figs. 4d, 5d, and 6d). This obviously indicates that MOLE and OEO have a direct potent immunostimulant effects and enhance the immune responses to NDV vaccine. The enhancement effects of MOLE on immune response to NDV vaccine was matched with the findings of (Younis et al. 2016; Younis and Elbestawy 2017; Eze et al. 2014) who indicated that supplementation of broiler chicks with *Moringa Oleifera* increased the antibody titers for New Castle Disease Virus. The antioxidant properties of *Moringa Oleifera* leaves extract can prevent the production of reactive oxygen species (ROS) and free radicals, which subsequently maintain the effective bird immunity (Sofidiya et al. 2006; Ogbunugafor et al. 2011). Similarly, oregano essential oil promotes faster growth and reduce the utilization of antibiotics in broiler chicks (Symeon et al. 2010) as it contains high concentrations of phytonutrients such antioxidants carotenoids, tocopherols, and ascorbic acid (Qwele et al*.* 2013; Saini et al. 2014).

## Conclusion

*Moringa Oleifera* alcoholic extract and Oregano essential oil improved broiler chicks’ growth and immune performance as they increased body weight, total leukocytic count, differential leukocytic count, phagocytic activity, and phagocytic index. In addition, they ameliorated cyclophosphamide reduced body weight and impaired immune response via increased body weight, total leukocytic count, differential leukocytic count, phagocytic activity, phagocytic index, hemagglutinin inhibition titer for New Castle disease virus, lymphoid organs proliferation, and reduced the mortality rate.

## Data Availability

All data used in this study is included in this published article.
